# Facilitating Evaluation of Hemolytic Uremic Syndrome Long-Term Health Outcomes Through Social Media Support Groups

**DOI:** 10.3389/fpubh.2020.544154

**Published:** 2020-11-23

**Authors:** Aaron T. E. Beczkiewicz, Robert L. Scharff, Barbara B. Kowalcyk

**Affiliations:** ^1^Department of Food Science and Technology, The Ohio State University, Columbus, OH, United States; ^2^Department of Human Sciences, The Ohio State University, Columbus, OH, United States

**Keywords:** hemolytic uremic syndrome, long-term health outcomes, posttraumatic stress (PTS), foodborne illness, burden of illness, cost of illness (COI)

## Abstract

Individual burden and cost of hemolytic uremic syndrome (HUS)—a medical condition characterized by acute kidney failure—can be substantial when accounting for long-term health outcomes (LTHOs). Because of the low incidence of HUS, evaluation of associated LTHOs is often restricted to physician and outbreak cohorts, both of which may not be representative of all HUS cases. This exploratory study recruited participants from private social media support groups for families of HUS cases to identify potential LTHOs and costs of HUS that are not currently measured. Additionally, this study sought to identify case characteristics that may confound or modify these LTHOs and costs of HUS. Respondents self-selected to complete an online cross-sectional survey on acute and chronic illness history, treatments, and public health follow-up for HUS cases. Posttraumatic stress among respondents (typically case parents) was also evaluated. Responses were received for 74 HUS cases from 71 families representing all geographic regions, and levels of urbanicity within the US self-reported symptoms were typical for HUS, while 35.1% of cases reported antibiotic treatment at any point during the acute illness. Hospital transfers were reported by 71.6% of cases introducing possible delays to care. More than 70% of cases reported experiencing at least one LTHO, with 45% of cases reporting renal sequelae. Posttraumatic stress symptoms were frequently reported by respondents indirectly affected by HUS. Potentially large economic costs that are not addressed in existing analyses were identified including both financial and more general welfare losses (lost utility). While biases in the study design limit the generalizability of results to all HUS cases, this study provides new insights into unmeasured LTHOs and costs associated with HUS. These results suggest that robustly designed cohort studies on HUS should include measures of psychosocial impacts on both the affected individual and their family members.

## Introduction

Hemolytic uremic syndrome (HUS) is a medical condition characterized by acute kidney failure resulting from hemolytic anemia and thrombocytopenia (1–3). HUS is commonly associated with Shiga toxin–producing *Escherichia coli* (STEC) infections, and 4–17% of STEC O157:H7 illnesses are estimated to progress to HUS (4). The incidence of HUS in the United States is relatively low with the Foodborne Diseases Active Surveillance Network (FoodNet) identifying 54 cases (0.49 cases per 100,000 persons) of pediatric HUS in 2017 (5). Despite the low incidence, the individual burden of HUS can be quite substantial. For example, HUS has an approximate 5% mortality rate, and renal and circulatory system sequelae are estimated to occur among 25% of HUS survivors (6, 7). The economic burden of HUS is estimated to be $541,695 per case (8), but many of the long-term health outcomes (LTHOs) associated with HUS (9, 10) have not been included in these estimates because of insufficient evidence. Thus, the individual burden of HUS may be substantially higher.

The potential for underestimating the burden of HUS is further heightened when considering potential LTHOs that are not currently identified or measured among cases. For example, there has been relatively little attention paid to potential psychosocial impacts of HUS on both the patient and their families. Given that HUS primarily affects young children, the 2017 incidence of HUS among children <5 years old (1.22 cases per 100,000 persons) was more than double that for all pediatric cases as previously cited—there are potentially substantial psychosocial ramifications for family members who are responsible for providing care to HUS survivors (5, 11). For example, a previous study of 30 HUS case–parent dyads in Scotland identified emotional and psychological distress, changes to daily behavior, and increased fear of the future among interviewed parents (12). Recognizing the limitations of the qualitative methods they used, Pollock et al. recommended measuring the psychosocial impact of HUS among families through diagnosis of posttraumatic stress disorder (PTSD) or another defined metric.

Identifying and evaluating the scope of LTHOs among HUS cases have been difficult, largely due to the low incidence. Consequently, studies on sequelae associated with HUS have been primarily restricted to physician cohorts, which are potentially biased toward higher severity cases needing more specialized care; cohorts established following large outbreaks, which may not be representative of sporadic HUS cases; and systematic reviews or meta-analyses of physician and outbreak cohorts (4). While a cohort study design is preferred for estimating prevalence and relative risk of LTHOs following acute illness, such studies are often resource-intensive and have small sample sizes, discouraging inclusion of additional exploratory data collection. In contrast, exploratory cross-sectional surveys of social media support communities offer a low-cost approach for discovering factors that should be further examined in cohort studies. Therefore, a cross-sectional survey of members of two HUS-related social media groups was undertaken to identify potential LTHOs and costs of HUS that are not currently measured. Additionally, this study sought to identify case characteristics that may confound or modify these LTHOs and costs of HUS as risk factors can be important in studies that extrapolate from samples to populations as a means of adjusting outcomes for population characteristics.

## Materials and Methods

This cross-sectional study recruited participants from two private Facebook support groups for families of individuals who developed HUS following an infectious diarrheal illness. No minimum *a priori* sample size was determined, and respondents (primarily HUS case parents) self-selected over a span of 1 month (February 2019) to complete an online survey developed and distributed by the research team using Qualtrics Online Survey Software (Provo, UT) (Supplementary Data Sheet 1). Because of the exploratory nature of this study and the limited pool of potential participants, the survey instrument was not validated prior to distribution. Within the survey, respondents were asked a series of questions on the acute illness, public health follow-up, chronic illnesses, treatments for HUS, and related sequelae experienced by the HUS case. Additionally, survey respondents were asked about posttraumatic stress (PTS) symptoms related to the HUS event. LTHOs were included within the survey if they have been previously reported in the literature as potential sequelae of foodborne infectious diseases including HUS, salmonellosis, campylobacteriosis, and yersiniosis among others (9). These LTHOs included intestinal disorders, hypertension, heart disease, chronic kidney disease, kidney transplant, surgery other than organ transplant, stroke, epilepsy or seizures, coma, bone disease (e.g., osteomyelitis) or skeletal deformities, impaired growth, and self-reported PTSD. Death following HUS was not included among outcomes evaluated to minimize risk of emotional stress on study participants while completing the survey. Questions evaluating PTS symptoms were adapted from a tool previously developed to assess PTSD symptoms aligning with the *Diagnostic and Statistical Manual of Mental Disorders* (5th edition) (Table 1) (13, 14). Respondents were classified as having PTS if they reported the minimum number of symptoms (one intrusion symptom, one avoidance symptom, two cognition and mood symptoms, and two arousal and reactivity symptoms) in all categories as required for clinical diagnosis of PTSD (NIMH 2020).

**Table 1:** Posttraumatic stress symptoms evaluated among survey respondents.

**Symptom category**	**Survey question^a^**
**DICHOTOMOUS (YES/NO) QUESTIONS**	
Intrusion	Have you experienced distressing memories about the HUS event?
Intrusion	Have you experienced bad dreams or nightmares related to the HUS event?
Intrusion	Have you ever felt like you were experiencing the time immediately leading up to or following the HUS event again?
Intrusion	Have reminders of the HUS event caused you to become very Emotionally upset (i.e., fear, sadness, anger, guilt or shame, worry, etc.)
Avoidance	Have you attempted to avoid thoughts or feelings related to the HUS event?
Avoidance	Have you attempted to make efforts to avoid activities, situations, or places that remind you of the HUS event or feel more dangerous since the HUS event?
Cognition and mood	Is there any aspect of the time frame immediately leading up to the HUS event that you cannot remember (i.e., gap in memory)?
Cognition and mood	Have you viewed yourself or the world in a more negative way since the HUS event?
Cognition and mood	Have you blamed yourself for the HUS event and the ensuing outcomes?
Cognition and mood	Since the HUS event, have you lost interest in activities you used to participate in?
Cognition and mood	Have you felt detached or cut off from others since the HUS event?
Cognition and mood	Have you had difficulty experiencing positive feelings?
Arousal and reactivity	Have you had difficulty concentrating?
Arousal and reactivity	Have you had difficulty falling or staying asleep?
**OPEN-ENDED TEXT RESPONSE**	
Avoidance	What activities, situations, or places do you avoid?
Avoidance	What do you do to try and avoid these activities?
Cognition and mood	What are some examples (of how you have viewed yourself or the world in a more negative way since the HUS event)?
Cognition and mood	What are some examples (of how you have had difficulty experiencing positive feelings)?
All symptoms	Are there any other ways in which your experience with HUS has changed or impacted your daily life or lifestyle in any way?

a *Posttraumatic stress symptom questions adapted from the PTSD Symptom Scale (13, 14)*.

Responses were classified and analyzed as a case if they reported that HUS was officially diagnosed by a medical provider. Sociodemographic factors, acute symptoms, clinical care, and LTHOs experienced by the HUS case and PTS experienced by respondents were summarized with descriptive statistics. Geographic location at time of illness was categorized according to US Census Bureau regional definitions. Urbanicity of residence at time of illness was categorized according to the National Center for Health Statistics 2013 urbanization definitions by zip code.

Logistic regression was used to explore whether report of any LTHO was higher as time (categorized as <1, 1 to <5, 5 to <10, ≥10 years) progressed since a case's HUS illness. Logistic regression was also used to explore potential risk factors for specific LTHOs among HUS cases and for PTS among respondents. Considered risk factors were case demographics, acute illness symptoms, treatment, and hospitalization history. All potential risk factors were included in the multivariable model selection process, which utilized a backward selection approach to estimate odds ratios adjusted (at minimum) for case age and gender. Qualitative analysis of open text response questions pertaining to PTS among respondents was performed, and recurring themes identified across respondents. Statistical analyses were performed using SAS 9.4 (Cary, NC). Appropriate ethical approvals were obtained from The Ohio State University Social and Behavioral Institutional Review Board (study #2018B0456).

## Results

### Acute Illness

**Figure 1:**
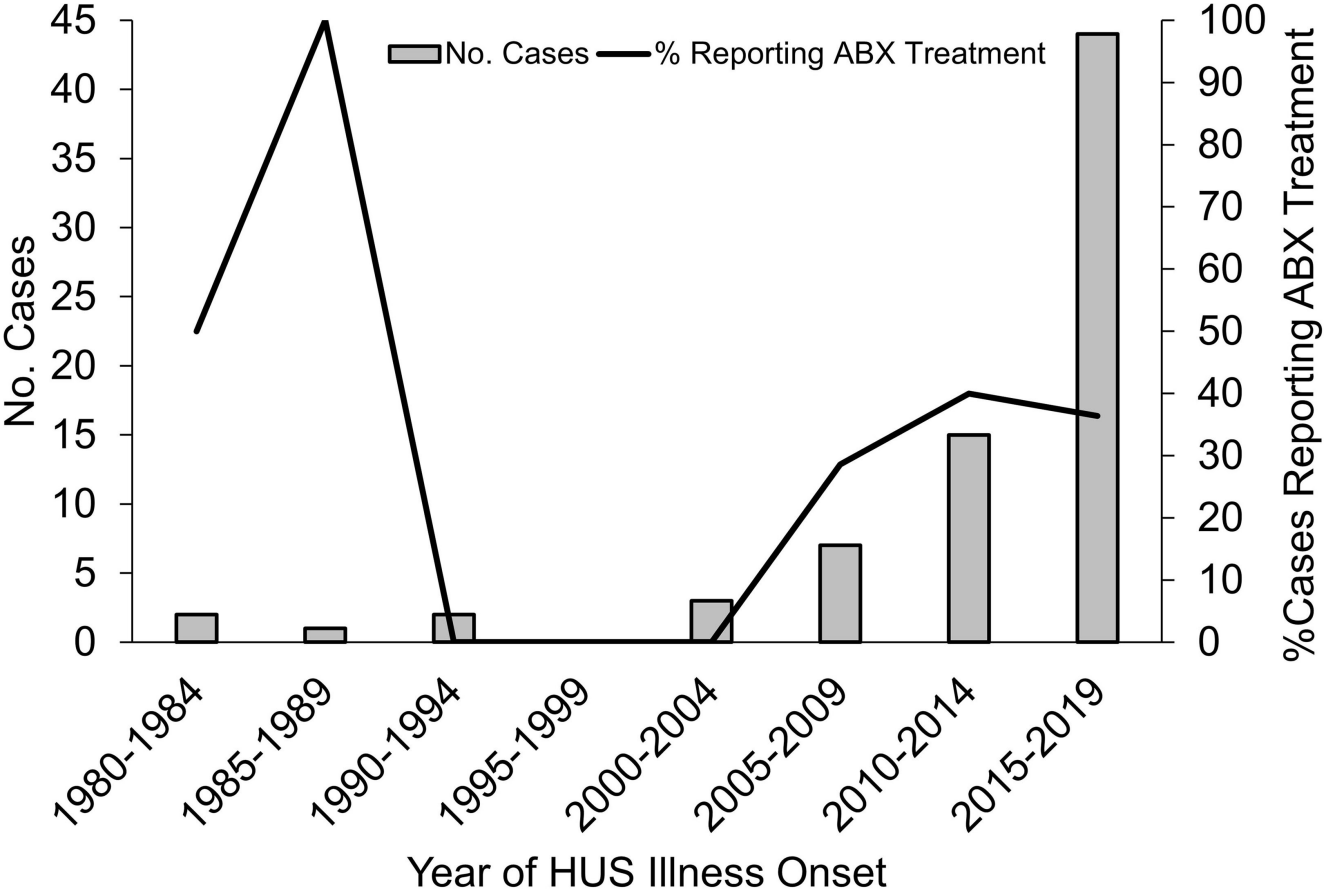
Self-reported antibiotic treatment among HUS cases by year of illness onset. HUS, hemolytic uremic syndrome; ABX, antibiotic; STEC, Shiga toxin–producing *E. coli*. Report of antibiotic treatment refers to treatment at any point during the acute illness phase and does not distinguish between treatment before and/or after diagnosis with STEC or HUS.

Surveys were completed for 84 illness events. Ten surveys were excluded from analyses because of no report of official HUS diagnosis by a medical provider resulting in a final study population of 74 HUS cases from among 71 families. The mean and median time elapsed between the HUS event and survey completion was 6.4 and 3.0 years, respectively (Table 2). All geographic regions and levels of urbanicity within the US were represented among the case population. More than 50% of cases developed HUS when they were younger than 5 years. Self-reported acute illness symptoms were typical for STEC and HUS, with a majority of individuals reporting bloody diarrhea and acute kidney failure (Table 3). A source of illness (e.g., contaminated food item) was identified for 19 (25.7%) of the 74 HUS cases. Interestingly, there was an increasing trend in antibiotic use with 35% of HUS cases reporting treatment with antibiotics at any point during the acute phase of illness (Figure 1).

**Table 2:** Self-reported demographic profile of HUS cases^a^.

**Case characteristic**		**No. cases (%)**
		**(*n* = 74)**
Gender	Male	30 (40.5)
	Female	44 (59.5)
Race and ethnicity	White, non-Hispanic	69 (93.2)
	Nonwhite and/or Hispanic	5 (6.8)
Age^b^ (illness onset) (years)	<5	40 (54.0)
	5 to <15	27 (36.5)
	15 to <30	5 (6.8)
	30 to <45	2 (2.7)
Time since HUS event^c^ (years)	<1	20 (27.0)
	1 to <5	24 (32.4)
	5 to <10	15 (20.3)
	≥10	15 (20.3)
Residence—geographic region^d^	Northeast	3 (4.1)
	Midwest	18 (24.3)
	South	17 (23.0)
	West	18 (24.3)
	Unknown	18 (24.3)
Residence—urbanicity^e^	Urban	42 (56.8)
	Rural	14 (18.9)
	Unknown	18 (24.3)

*HUS, hemolytic uremic syndrome*.

a *Case defined as individual who experienced medical provider diagnosed HUS*.

b *Median age = 4.0 years, mean age = 6.1 years*.

c *Median time since HUS = 3.0 years, mean time since HUS = 6.4 years*.

d *According to US Census Bureau regional definitions*.

e *According to US National Center for Health Statistics 2013 urbanization definitions*.

**Table 3:** Self-reported acute illness characteristics of HUS cases^a^.

**Case characteristic**		**No. cases (%)^b^**
		**(*n* = 74)**
Acute symptoms	Diarrhea	67 (90.5)
	Bloody diarrhea	55 (74.3)
	Nausea	49 (66.2)
	Vomiting	58 (78.4)
	Fever	40 (54.1)
	Acute kidney failure	68 (91.9)
Treatment for acute illness	Antibiotic	26 (35.1)
	Antidiarrheal	12 (16.2)
	Blood transfusion	66 (89.2)
	Dialysis	60 (81.1)
	Other surgery	14 (18.9)
Hospital facility type^c^	Urban hospital	22 (29.7)
	Rural hospital	4 (5.4)
	Pediatric specialty hospital	59 (79.7)
	Teaching hospital	22 (29.7)
Acute illness investigation	Specimen collected	67 (90.5)
	Source of illness identified^d^	19 (25.7)
	Follow-up by health department	54 (73.0)

a *Case defined as individual who was diagnosed with HUS by a medical provider*.

b *Respondents could select more than one response and so may not add up to 100%*.

c *All 74 cases were hospitalized. Multiple hospital types were possible per case due to hospital transfer*.

d *Confirmed sources of illness include petting zoo or fairground (n = 3), ground beef (n = 2), raw milk (n = 2), and spinach (n = 1)*.

Cases were predominantly treated at pediatric specialty hospitals, with 53 cases (71.6%) being transferred to a different hospital during their acute illness. Self-reported reasons for hospital transfer (with some cases reporting multiple reasons) included need for pediatric specialist by 26 transferred cases (49.1%), need for renal specialist by 15 transferred cases (28.3%), need for dialysis unavailable at initial facility for 12 transferred cases (23.6%), rural or small hospital unable to provide necessary care for six transferred cases (11.3%), and medical provider at initial location unsure of diagnosis and/or treatment for HUS for four transferred cases (7.5%).

### Long-Term Health Outcomes

At least one LTHO was reported by 52 cases (70.3%), with renal sequelae (hypertension and chronic kidney disease) being the most commonly reported (Table 4). Length of time between HUS event and survey completion (<1, 1 to <5, 5 to <10, ≥10 years) was not significantly associated with report of any LTHO. The small number of cases in the 5+ years postillness groups hindered the evaluation of potential associations between time since illness and LTHOs. Univariable analyses identified associations between acute illness factors and chronic kidney failure, surgery other than transplant, bone disease (e.g., osteomyelitis), and self-reported PTSD (Table 5). Complete univariable analyses for LTHO risk factors are available online (Supplementary Table 1). The multivariable model for chronic kidney failure identified associations with urbanicity, acute dialysis, and hospital transfer (adjusting for case age and gender); the multivariable model for self-reported PTSD identified associations with case age and hospital transfer (adjusting for case gender) (Table 6). There were no significant factors for surgery other than transplant and bone disease in the multivariable model.

**Table 4:** Report of long-term health outcomes among HUS cases by length of time since acute illness.

	**No. cases reporting LTHO (%)**				
**LTHO**	**All cases**	**<1 year PI**	**1 to <5 years PI**	**5 to <10 years PI**	**≥10 years PI**
	**(*n* = 74)**	**(*n* = 20)**	**(*n* = 24)**	**(*n* = 15)**	**(*n* = 15)**
**NUMBER OF LTHOs**^a^					
0 LTHOs	22 (29.7)	9 (45.0)	7 (29.2)	2 (13.3)	4 (26.7)
≥1 LTHOs	52 (70.3)	11 (55.0)	17 (70.8)	13 (86.7)	11 (73.3)
**SELF-REPORTED LTHO**					
Intestinal disorders	15 (20.3)	2 (10.0)	6 (25.0)	4 (26.7)	3 (20.0)
Hypertension	35 (47.3)	10 (50.0)	13 (54.2)	6 (40.0)	6 (40.0)
Heart disease	5 (6.8)	0 (0.0)	2 (8.3)	3 (20.0)	0 (0.0)
Chronic kidney disease	32 (43.2)	8 (40.0)	7 (29.2)	12 (80.0)	5 (33.3)
Kidney transplant	5 (6.8)	0 (0.0)	0 (0.0)	1 (6.7)	4 (26.7)
Other surgery	5 (6.8)	1 (5.0)	1 (4.2)	1 (6.7)	2 (13.3)
Stroke	4 (5.4)	2 (10.0)	0 (0.0)	2 (13.3)	0 (0.0)
Epilepsy or seizures	1 (1.4)	1 (5.0)	0 (0.0)	0 (0.0)	0 (0.0)
Coma	1 (1.4)	1 (5.0)	0 (0.0)	0 (0.0)	0 (0.0)
Bone disease	3 (4.1)	0 (0.0)	0 (0.0)	2 (13.3)	1 (6.7)
Impaired growth	6 (8.1)	0 (0.0)	2 (8.3)	2 (13.3)	2 (13.3)
PTSD	16 (21.6)	3 (15.0)	6 (25.0)	5 (33.3)	2 (13.3)

*LTHOs, long-term health outcomes; HUS, hemolytic uremic syndrome; PI, post illness; PTSD, posttraumatic stress disorder*.

a *Mean no. LTHOs per case (standard deviation) = 1.7 (1.6), range no. LTHOs per case = 0 – 7*.

**Table 5:** Significant univariable logistic regression associations between risk factors and long-term health outcomes following HUS^a^.

		**No. with LTHO**		
**Self-reported LTHO**	**Risk factor**	**Factor present (%)**	**Factor absent (%)**	**Odds ratio (95% CI)**
PTSD	Age <5 years	5/40 (12.5)	11/34 (32.4)	0.30 (0.092, 0.973)
Bone disease	Antidiarrheal treatment	2/12 (16.7)	1/62 (1.6)	12.20 (1.010, 147.420)
Chronic kidney failure	Dialysis	30/60 (50.0)	2/14 (14.3)	6.00 (1.236, 29.135)
	Hospital transfer	27/53 (50.9)	5/21 (23.8)	3.32 (1.063, 10.385)
Surgery other than transplant	Pediatric hospitalization	2/59 (3.4)	3/15 (20.0)	0.14 (0.021, 0.933)
	Urban hospitalization	4/22 (18.2)	1/52 (1.9)	11.33 (1.187, 108.199)
	Surgery during acute HUS illness	3/14 (21.4)	2/60 (3.3)	7.91 (1.181, 52.972)

*LTHO, long-term health outcome; CI, confidence interval*.

a *Univariable associations significant at α = 0.05. Complete analyses are available online (Supplementary Table 1)*.

**Table 6:** Multivariable logistic regression associations between risk factors and long-term health outcomes following HUS.

**Self-reported LTHO**	**Risk factor**	**Adjusted odds ratio (95% CI)**	**χ^2^*p*^a^**
Kidney failure	Age <5 years	2.35 (0.746–7.399)	0.144
	Female	1.88 (0.622–5.686)	0.263
	**Dialysis**	**10.41 (1.812–59.778)**	**0.009**
	**Hospital transfer**	**4.13 (1.143–14.938)**	**0.030**
	**Residence—rural**	**0.19 (0.040–0.901)**	**0.037**
PTSD	**Age**<**5 y**	**0.18 (0.051–0.666)**	**0.010**
	Female	0.53 (0.154–1.803)	0.307
	**Hospital transfer**	**5.39 (1.011–28.746)**	**0.049**

*LTHO, long-term health outcome; CI, confidence interval; PTSD, posttraumatic stress disorder*.

a *Bold text indicates significant association (α = 0.05)*.

### Posttraumatic Stress and Costs of Illness

Stress symptoms were reported by 69 survey respondents (97.2%) (Table 7). Twenty respondents (28.2%) reported the minimum number of symptoms across all categories and met this study's definition of PTS. Among potential risk factors evaluated, only case age younger than 5 years was marginally associated (α = 0.10) with PTS among survey respondents (Table 8). Controlling for other case acute factors such as gender, race, and ethnicity and urbanicity did not significantly improve the model fit for PTS and case age.

**Table 7:** Self-reported posttraumatic stress symptoms among survey respondents (*n* = 71)^a^.

**Symptom category**	**Mean no. symptoms (sd)**	**Min. required no. symptoms^b^**	**No. respondents meeting category min. (%)**
Intrusion	3.6/5.0 (1.3)	1	69 (97.2)
Avoidance	1.4/2.0 (0.7)	1	60 (84.5)
Cognition and mood	2.3/6.0 (1.8)	2	44 (62.0)
Arousal and reactivity	1.0/2.0 (0.9)	2	26 (36.6)

*sd, standard deviation; Min, minimum; PTSD, posttraumatic stress disorder*.

a *Posttraumatic stress symptom questions adapted from the PTSD Symptom Scale (13, 14). Complete set of questions asked of respondents are reported in Table 1 and online (Supplementary Data Sheet 1)*.

b *Minimum number of symptoms within each category defined as the number of symptoms per category required for clinical diagnosis of PTSD (NIMH 2020)*.

**Table 8:** Univariable logistic regression associations between acute risk factors and posttraumatic stress among respondents following an HUS event.

	**No. respondents with PTS^a^**			
**HUS case risk factor**	**Factor present (%)**	**Factor absent (%)**	**Odds ratio (95% CI)**	**χ^2^*p*^b^**
**Age**<**5 y**	**15/40 (37.5)**	**6/34 (17.7)**	**2.80 (0.942–8.325)**	**0.064**
Female	10/44 (22.7)	11/30 (36.7)	0.51 (0.182–1.414)	0.195
Nonwhite and/or Hispanic	3/5 (60.0)	18/69 (26.1)	4.25 (0.656–27.524)	0.129
Residency in rural area	2/14 (14.3)	19/60 (31.7)	0.36 (0.073–1.768)	0.208
Acute kidney failure	19/68 (27.9)	2/6 (33.3)	0.78 (0.131–4.590)	0.779
Transfusion	19/66 (28.8)	2/8 (25.0)	1.21 (0.225–6.551)	0.823
Dialysis	16/60 (26.7)	5/14 (35.7)	0.65 (0.191–2.248)	0.501
Surgery during acute HUS illness	6/14 (42.9)	15/60 (25.0)	2.25 (0.672–7.538)	0.189
Pediatric hospitalization^c^	16/59 (27.1)	5/15 (33.3)	0.74 (0.220–2.514)	0.634
University/teaching hospitalization^c^	8/22 (36.4)	13/52 (25.0)	1.71 (0.587–5.006)	0.324
Rural hospitalization^c, d^	0/4 (0.0)	21/70 (30.0)		1.000
Urban hospitalization^c^	8/22 (36.4)	13/52 (25.0)	1.71 (0.587–5.006)	0.324
Transferred to different hospital	15/53 (28.3)	6/21 (28.6)	0.99 (0.322–3.023)	0.982

*PTS, posttraumatic stress; CI, confidence interval; HUS, hemolytic uremic syndrome*.

a *PTS defined as experiencing the minimum number of symptoms per category (intrusion, avoidance, cognition and mood, arousal, and reactivity) in all categories required for clinical diagnosis of posttraumatic stress disorder (NIMH 2020)*.

b *Bold text indicates marginally significant association (α = 0.10)*.

c *More than one type of hospitalization possible if HUS case transferred to different hospital*.

d *Unable to estimate association of PTS with rural hospitalization due to separation of data*.

Situations, places, and activities avoided by respondents following the HUS event included outdoor activities (e.g., camping, swimming), agritourism (e.g., petting zoos, farms, fairs, and festivals), foods and/or locations associated with contracting the illness, and medical facilities frequented for clinical care during and after the HUS illness. Ways in which respondents viewed themselves or the world more negatively since the HUS event were synthesized into recurring themes of (1) distrust of others (e.g., agriculture and food industries, government agencies, and medical professionals), (2) fear and anxiety for future medical needs of HUS survivors, and (3) blaming oneself for a dependent child becoming ill. Selected open-ended responses demonstrating potential costs of HUS include the following:

- adjusting work (healthcare setting) responsibilities to avoid eliciting memories of HUS event
- discontinuation of work to provide long-term care for HUS survivor
- parental use of medical leave for depression following an HUS event
- postponing medical care unrelated to HUS due to fear and avoidance of healthcare facilities
- disruption of childhood education of HUS survivor due to LTHO management.

## Discussion

This work demonstrates that social media–based support communities can serve as a low-cost means of identifying potential burdens that have previously been overlooked in cohort studies of HUS and other low-incidence infectious diseases. While previous studies have quantified the economic burden from HUS based on direct medical expenses, productivity losses, and deaths (8, 15), this study has identified potentially large economic costs that are not currently addressed in cohort studies of HUS and existing burden analyses. These unmeasured economic costs include both financial and more general welfare losses (lost utility). Missing financial costs include travel costs associated with hospital transfers, a better accounting of caregiver costs from lost productivity, and some medical costs associated with resulting LTHOs. Perhaps more importantly, assessments of lost welfare from psychosocial impacts associated with HUS are notably absent in the literature. Specifically, HUS-related costs have not been estimated for mental health services, productivity losses due to PTS induced presenteeism and absenteeism, and other avoidance effects. A high probability (28.2%) of PTS in this admittedly self-selected sample suggests that these costs could be very high. The financial cost per patient for major depressive disorder, for example, has been estimated to be $5,988 annually (16). Additionally, there are substantial utility losses for those affected by HUS. Both patients and family members suffer from these losses during treatment and, in many cases, long afterward. Having a better understanding of the total economic burden associated with HUS is important, both as a means of understanding how to provide sufficient care for families affected by the illness and as a means of directing scarce public health resources toward a problem that has, to this point, been undervalued.

This work also demonstrates the utility of social media–based support groups for identifying research priorities that warrant additional consideration. For example, it was surprising that 35% of cases in this study were treated with antibiotics during their acute illness (Figure 1). Despite conflicting evidence on associations between antibiotic treatment of STEC infections and risk of developing HUS (17), the US Centers for Disease Control and Prevention (CDC) recommends against use of antibiotics in the treatment of STEC infections (CDC 2019). This study was not able to determine whether antibiotics were prescribed empirically before or after diagnosis of either STEC or HUS. It is also important to note that these results should be interpreted with caution as the cross-sectional design and use of self-reported treatments may introduce recall bias. Still, it can be inferred that the cases identified by this study are likely to be representative of typical HUS cases in the United States given that self-reported symptoms and LTHOs aligned with those commonly reported by HUS cases (1–3). Additionally, the study was able to recruit a participant population similar in demographic profile to that of pediatric HUS cases ascertained by the FoodNet active surveillance system from 2000 to 2010 (18), and cases were enrolled from all geographic regions and levels of urbanicity within the United States. This suggests that, while this study was not designed for robust estimation, unmeasured outcomes or surprising results identified by this study are likely present, to some degree, within the population and require further consideration in studies capable of quantifying risk. Consequently, further exploration of this specific phenomenon is warranted to understand whether these results are indicative of broader prescribing practices.

Through exploratory risk factor analyses, this study also identified case characteristics that may confound or modify LTHOs and costs of HUS, which would be important for cohort studies seeking to adjust estimates when extrapolating from samples to populations. Specifically, adjusted odds of chronic kidney failure and self-reported PTSD were higher for patients who were transferred to a new facility compared to those who were not. This is not surprising given that hospital transfer may be a surrogate for more severe acute illness and driven by the need for dialysis, which, as seen in this study, has been associated with increased risk of chronic kidney failure among HUS cases (19). For example, 3 years after a German *E. coli* O104:H4 outbreak associated with sprouts, 32 of 72 (44%) HUS cases had renal sequelae, and dialysis during acute illness was associated with increased risk of renal sequelae (20). Adjusted odds of chronic kidney failure in this study were also lower among urban cases compared to rural cases. Several studies have shown that rural populations have higher exposure to STEC (21, 22) and consequently are at higher risk of HUS (23), although these results are not consistent with other studies (24). While sample size (74 HUS cases from among 71 families) likely limited this study to detecting only large effects, it is notable that the sample size in this study is larger than most cohort studies on LTHOs of HUS which frequently consist of <50 cases (4). Even so, the univariable and multivariable associations reported by this study between risk factors and LTHOs should not be interpreted as true population estimates. Instead, these results should be viewed as justification for inclusion of the associated case characteristics (e.g., residence urbanicity, type of hospital, history of hospital transfer) in future research on LTHOs of HUS due to their potential to confound or modify estimates.

There are several limitations that affect the generalizability of these results. The primary limitation of this study was self-selection bias, which is inherent in using an online survey with self-reported symptoms. Specifically, individuals with more severe LTHOs may have been more likely to either join a social media support group and/or respond to this survey, especially if experiencing PTS symptoms and/or LTHOs. This could lead to overestimation if the results were used to quantify burden directly. Because the objective of this study was to identify unmeasured outcomes, a greater likelihood of identifying extreme outcomes due to self-selection bias becomes a strength. The other key limitation of this work is the potential for recall bias. The majority of HUS cases identified by this study occurred within the 5 years leading up to completing the survey, which would be expected to decrease the impact recall bias. However, there may not be an easily definable relationship between time since HUS event and recall bias since PTS—which is characterized by both avoidance and intrusion symptoms—was identified among a large portion of respondents. If an individual experiences more avoidance symptoms, there could be greater recall bias due to subconscious suppression of memories following the HUS event. In contrast, if an individual experiences more intrusion symptoms, the impact of recall bias could be minimized due to frequent recollection of the HUS event. While the present study was unable to investigate further, future exploration of the impact of recall bias on recollection of an HUS event is warranted, given this divergence in recall would extend beyond retrospective social media–based studies to also impact cohort studies and surveillance activities.

While there are limitations inherent to this cross-sectional study, it is important to note that the limitations faced by this study do not conflict with the overarching goal, which was to identify LTHOs and costs of HUS for closer examination in future cohort studies. These results serve as useful starting point for more robust work estimating psychosocial costs to families of HUS cases. With approximately 30% of respondents experiencing PTS, a robustly designed prospective study that is capable of addressing the limitations discussed above is warranted to quantify prevalence and scope of LTHOs of HUS including PTS for integration in future iterations of burden and cost of illness estimation.

## Data Availability Statement

The raw data supporting the conclusions of this article will be made available by the authors, without undue reservation.

## Ethics Statement

The studies involving human participants were reviewed and approved by Behavioral and Social Sciences IRB at The Ohio State University. Written informed consent from the participants' legal guardian/next of kin was not required to participate in this study in accordance with the national legislation and the institutional requirements.

## Author Contributions

AB and BK conceived of and designed the study. AB coordinated data collection, performed statistical analyses, and wrote the first draft of the manuscript. RS and BK wrote sections of the manuscript and contributed to interpretation of data for the work. All authors contributed to manuscript revision, read and approved the submitted version.

## Conflict of Interest

AB and RS report no relationships that might be perceived by the academic community as representing a potential conflict of interest. BK, the study principal investigator, had a child who died from HUS in 2001 and is a member of the relevant private social media groups. BK did not participate in the study and was blinded to participant responses.
