# Supplementary material for: Facilitating Evaluation of Hemolytic Uremic Syndrome Long-Term Health Outcomes Through Social Media Support Groups
Source: Front Public Health. 2020 Nov 23;8:544154. doi: 10.3389/fpubh.2020.544154 (PMC7719744; doi:10.3389/fpubh.2020.544154)
Supplement: Supplementary file 2 [file Data_Sheet_1.PDF]

Supplementary Material A. Survey of Hemolytic Uremic Syndrome Case Families
The Ohio State University – February 2019

Start of Block: Consent to Participate

Q0 **The Ohio State University**
Consent to Participate in Research
Study Title: Impacts of Hemolytic Uremic Syndrome (HUS)
Principal Investigator: Dr. Barbara Kowalcyk
Sponsor: NONE
This is a consent form for research participation. It contains important information about this study and what to expect if you decide to participate.
Your participation is voluntary. Please consider the information carefully. Feel free to ask questions before making your decision whether or not to participate.
Purpose:
This study is being conducted to explore the impact of HUS on those both directly and indirectly affected by Hemolytic Uremic Syndrome (HUS). Additionally, this study seeks to identify risk factors that may be of interest to future research on the epidemiology of HUS.
Procedures/Tasks:
Participants in this study will complete a survey pertaining to your experience with HUS, the impact it has had on your life, sociodemographic background, and medical and/or genetic factors that may be of interest to future research.
The survey is expected to take approximately 30 minutes to complete.
Duration:
You may discontinue the survey at any time. If you decide to discontinue the survey, there will be no penalty to you, and you will not lose any benefits to which you are otherwise entitled. Your decision will not affect your future relationship with The Ohio State University.
Risks and Benefits:
There is no more than minimal risk if you choose to participate; however, there is potential for the survey questions to illicit emotional distress through recall of traumatic events. If completion of this survey will negatively impact any aspect of your health or well-being, please do NOT begin the survey.
There are no individual benefits to participation in this study.
Confidentiality:
We will work to make sure that no one sees your online responses without approval. But, because we are using the Internet, there is a chance that someone could access your online responses without permission. In some cases, this information could be used to identify you. Also, there may be circumstances where this information must be released. For example, personal information regarding your participation in this study may be disclosed if required by state law. Also, your records may be reviewed by the following groups (as applicable to the research): Office for Human Research Protections or other federal, state, or international regulatory agencies; The Ohio State University Institutional Review Board or Office of Responsible Research Practices; The sponsor, if any, or agency (including the Food and Drug Administration for FDA-regulated research) supporting the study.
Incentives:
There are no incentives to participate in this survey.
Participant Rights:
You may refuse to participate in this study without penalty or loss of benefits to which you are otherwise entitled. If you are a student or employee at Ohio State, your decision will not affect your grades or employment status.
If you choose to participate in the study, you may discontinue participation at any time without penalty or loss of benefits. By agreeing to participate, you do not give up any personal legal rights you may have as a participant in this study.

Supplementary Material A. Survey of Hemolytic Uremic Syndrome Case Families
The Ohio State University – February 2019

An Institutional Review Board responsible for human subjects research at The Ohio State University reviewed this research project and found it to be acceptable, according to applicable state and federal regulations and University policies designed to protect the rights and welfare of participants in research.

Personal Identifiers in Survey:
This survey collects the following information that may potentially be used to identify you:

First 3 letters of last name   First letter of first name   ZIP code at time of HUS event

Potential identifiers will be used to ensure accuracy of data and determine type of residential location (i.e., urban, rural) at the time of the HUS event. These potential identifiers will be removed by the study coordinator following this step.
Personal identifiers will not be reused or disclosed to any other person or entity (except as required by law for authorized oversight of the research). Additionally, identifiers will not be used for other research for which disclosure of these identifiers is permitted.

Contacts and Questions:
For questions, concerns, or complaints about the study, or if you feel you have been harmed as a result of study participation, you may contact the principal investigator or study coordinator respectively:

Barbara Kowalyck, PhD, MA
Assistant Professor
The Ohio State University
Parker Food Science Building
2015 Fyffe Road
Columbus, OH 43210
kowalcyk.1@osu.edu

Aaron Beczkiewicz, MPH
Graduate Research Associate
The Ohio State University
beczkiewicz.1@osu.edu

For questions about your rights as a participant in this study or to discuss other study-related concerns or complaints with someone who is not part of the research team, you may contact the Office of Responsible Research Practices at 1-800-678-6251 or hsconcerns@osu.edu.

Providing consent
I have read (or someone has read to me) this page and I am aware that I am being asked to participate in a research study personally and/or as the legal guardian of a minor. I have had the opportunity to ask questions and have had them answered to my satisfaction. I voluntarily agree to participate in this study. I am not giving up any legal rights for myself and/or any minors I may be completing this survey for by agreeing to participate.

To print or save a copy of this page, select the print button on your web browser.

Please click the button below to proceed and participate in this study. If you do not wish to participate, please close out your browser window.

End of Block: Consent to Participate

Supplementary Material A. Survey of Hemolytic Uremic Syndrome Case Families
The Ohio State University – February 2019

Start of Block: Determining role

Q1 What is your relationship to an individual who has been diagnosed with HUS? (select all that apply)

- Self
- Parent
- Child
- Sibling
- Spouse
- Other (specify) ____

Q1.1 How were you made aware of this survey?

- Social media
- Health clinic
- Law firm
- Personal referral from another individual
- Other (specify) ____

End of Block: Determining role

Start of Block: Sociodemographic

Q2 How many individuals are in your family?

____

Q3 What was your family's US postal ZIP code at the time of initial HUS illness? If your family resided outside the US, enter 99999.

____

Q4 How many individuals in your family experienced diarrheal illness?

____

Q5 How many individuals in your family were diagnosed with a Shiga toxin-producing *E. coli* (STEC) infection?

____

Q6 How many individuals in your family were diagnosed with HUS?

____

Supplementary Material A. Survey of Hemolytic Uremic Syndrome Case Families
The Ohio State University – February 2019

Q7 Are any family members of Hispanic, Latino or Spanish origin?

	Hispanic, Latino, or Spanish origin?			
	Yes	No	Decline to answer	Not applicable
HUS case				
Parent(s) of HUS case				
Spouse of HUS case				
Sibling(s) of HUS case				
Grandparent(s) of HUS case				

Q8 Which of the following best describes the race of individuals in your family? (select all that apply)

	Race					
	White	Black or African American	Asian or Pacific Islander	Other	Decline to answer	Not applicable
HUS case						
Parent(s) of HUS case						
Spouse of HUS case						
Sibling(s) of HUS case						
Grandparent(s) of HUS case						

Supplementary Material A. Survey of Hemolytic Uremic Syndrome Case Families
The Ohio State University – February 2019

Q9 Do any family members experience the following preexisting conditions? (select all that apply)

	Preexisting conditions									
	Diabetes	Heart disease	Hematologic disease (i.e., blood disorder)	Cancer	Immunosuppressive condition or immunodeficiency	Liver disease	Kidney disease	Other	Decline to answer	Not applicable
HUS case										
Parent(s) of HUS case										
Spouse of HUS case										
Sibling(s) of HUS case										
Grandparent(s) of HUS case										

Supplementary Material A. Survey of Hemolytic Uremic Syndrome Case Families
The Ohio State University – February 2019

Q10 Which of the following ABO blood groups are represented within your family? (select all that apply)

	ABO blood group									
	A positive	A negative	B positive	B negative	AB positive	AB negative	O positive	O negative	Unknown	Not applicable
HUS case										
Parent(s) of HUS case										
Spouse of HUS case										
Sibling(s) of HUS case										
Grandparent(s) of HUS case										

Q11 Which of the following eye colors are represented within your family? (select all that apply)

	Eye color					
	Blue	Green	Brown	Other	Unknown	Not applicable
HUS case						
Parent(s) of HUS case						
Spouse of HUS case						
Sibling(s) of HUS case						
Grandparent(s) of HUS case						

Q12 Which of the following natural hair colors are represented within your family? (select all that apply)

	Hair color						
	Blonde	Brown	Black	Red	Other	Unknown	Not applicable
HUS case							
Parent(s) of HUS case							
Spouse of HUS case							
Sibling(s) of HUS case							
Grandparent(s) of HUS case							

End of Block: Sociodemographic

Supplementary Material A. Survey of Hemolytic Uremic Syndrome Case Families
The Ohio State University – February 2019

Start of Block: History - logic

L1 The following set of questions pertains to acute and ongoing illness history. At the end of the section, you will be prompted to either "Complete illness history for another individual" or "Continue with the rest of the interview". Begin by completing the form for yourself regardless of whether you were ill or not.

End of Block: History - logic

Start of Block: History - Self

S13 What are the first 3 letters of your last name?

____

S14 What is the first letter of your first name?

____

S15 In what year were you born?
Birth Year

DROPDOWN MENU

S16 What is your gender?
- Male
- Female
- Other
- Decline to answer

SX Did you experience any gastrointestinal illness or HUS?
- Yes
- No

Skip To: End of Block If Did you experience any gastrointestinal illness or HUS? = No

S17 When did your illness begin?
Year
Month

DROPDOWN MENU

S18 Which of the following signs and symptoms of illness did you experience at the time of your illness?

- Diarrhea
- Bloody diarrhea
- Nausea
- Vomiting
- Fever
- Kidney failure
- Other (specify) ____
- None

S19 Were you treated in any of the following ways?

- Antibiotic
- Antidiarrheal
- Dialysis
- Blood transfusion
- Kidney transplant
- Other surgery (specify) ____
- Other (specify) ____
- None

S20 Was a stool specimen collected?

- Yes
- No

Display Question S20A:
If Was a stool specimen collected? = Yes

S20A Was the stool specimen laboratory confirmed as positive for Shiga toxin-producing *E. coli* (STEC)?

- Yes
- No

S21 Was a source of the illness identified (i.e., food or environmental source)?

- Yes
- No
- Unknown

S22 Was the identity of the source of illness laboratory confirmed (i.e., same strain isolated from food item)?
- Yes
- No
- Unknown

Display Question S22A:
If Was the identity of the source of illness laboratory confirmed (i.e., same strain isolated from f... = Yes

S22A What was the identified source?

____

S23 Were you diagnosed with Hemolytic Uremic Syndrome (HUS)?
- Yes
- No

Display Question S23A:
If Were you diagnosed with Hemolytic Uremic Syndrome (HUS)? = Yes

S23A How was HUS diagnosed?
- Blood
- Urine
- Stool
- Other (specify) ____
- Not tested
- Unknown

S24 Were you hospitalized for your illness?
- Yes
- No

Skip To: S25 If Were you hospitalized for your illness? = No

S24A Which of the following best describe the facility in which you were hospitalized? (select all that apply)
- Urban hospital
- Rural hospital
- Pediatric specialty hospital
- Teaching hospital (associated with a university)
- Other (specify) ____
- Unknown

S24B Were you transferred from the admitting hospital to a different facility at any time during your illness?
- Yes
- No

Display Question S24Bi:
If Were you transferred from the admitting hospital to a different facility at any time during your... = Yes

S24Bi Was there a specific reason for your hospital transfer?

____
____
____
____
____

S24C Were you hospitalized in an Intensive Care or Critical Care Unit (ICU/CCU)?
- Yes
- No
- Unknown

S24D How long (days) was your hospital stay?

____

S25 Were you contacted by a health department following your illness?
- Yes
- No

S26 Was an exposure questionnaire completed?
- Yes
- No

Display Question S26A:
If Was an exposure questionnaire completed? = Yes

S26A Who administered the questionnaire?
- Self
- Medical provider
- Health department
- Other (specify) ____

S27 Was there any other follow-up by a health department not previously discussed? If so, please describe.

____
____
____
____
____

S28 Which of the following conditions have occurred following your illness with HUS that were not previously occurring.

	Time since HUS				Was this condition formally diagnosed by a medical provider?			Are you currently taking medications for this condition?			Are you currently seeing a doctor for this condition?		
	6 months	1 year	5 year	10 year	Yes	No	Not Applicable	Yes	No	Not Applicable	Yes	No	Not Applicable
Intestinal problems (i.e., irritable bowel disease, inflammatory colitis, etc.)													
High blood pressure													
Heart disease													
Kidney failure (specify current % kidney function)													
Kidney transplant													
Other surgery (specify)													
Stroke													
Epilepsy / seizures													
Coma													
Bone disease or skeletal deformities													
Impaired growth													
Post-traumatic stress disorder													
Other (specify)													

End of Block: History - Self

Start of Block: History - Proxy

L2 You are now completing illness histories for other individuals in your family who EXPERIENCED gastrointestinal illness or HUS.
An illness history can be completed for a maximum of 5 other individuals.

O1 Ill Individual 1

O1-13 What are the first 3 letters of the individual's last name?

____

O1-14 What is the first letter of the individual's first name?

____

O1-15 In what year was the individual born?
Birth Year

DROPDOWN MENU

O1-16 What is the individual's gender?
- Male
- Female
- Other
- Decline to answer

O1-17 When did the individual's illness begin?
Year
Month

DROPDOWN MENU

O1-18 Which of the following signs and symptoms of illness did the individual experience at the time of their illness?
- Diarrhea
- Bloody diarrhea
- Nausea
- Vomiting
- Fever
- Kidney failure
- Other (specify) ____
- None

Supplementary Material A. Survey of Hemolytic Uremic Syndrome Case Families
The Ohio State University – February 2019

O1-19 Was the individual treated in any of the following ways?
- Antibiotic
- Antidiarrheal
- Dialysis
- Blood transfusion
- Kidney transplant
- Other surgery (specify) ____
- Other (specify) ____
- None

O1-20 Was a stool specimen collected?
- Yes
- No

Display Question O1-20A:
If Was a stool specimen collected? = Yes

O1-20A Was the stool specimen laboratory confirmed as positive for Shiga toxin-producing *E. coli* (STEC)?
- Yes
- No

O1-21 Was a source of the illness identified (i.e., food or environmental source)?
- Yes
- No
- Unknown

O1-22 Was the identity of the source of illness laboratory confirmed (i.e., same strain isolated from food item)?
- Yes
- No
- Unknown

Display Question O1-22A
If Was the identity of the source of illness laboratory confirmed (i.e., same strain isolated from f... = Yes

O1-22A What was the identified source?

____

O1-23 Was the individual diagnosed with Hemolytic Uremic Syndrome (HUS)?
- Yes
- No

Display QuestionO1-23A:
If Was the individual diagnosed with Hemolytic Uremic Syndrome (HUS)? = Yes

Supplementary Material A. Survey of Hemolytic Uremic Syndrome Case Families
The Ohio State University – February 2019

O1-23A How was HUS diagnosed?
- Blood
- Urine
- Stool
- Other (specify) ____
- Not tested
- Unknown

O1-24 Was the individual hospitalized for their illness?
- Yes
- No

Skip To: O1-25 If Was the individual hospitalized for their illness? = No

O1-24A Which of the following best describe the facility in which the individual was hospitalized? (select all that apply)
- Urban hospital
- Rural hospital
- Pediatric specialty hospital
- Teaching hospital (associated with a university)
- Other (specify) ____
- Unknown

O1-24B Was the individual transferred from the admitting hospital to a different facility at any time during their illness?
- Yes
- No

Display Question O1-24B:
If Was the individual transferred from the admitting hospital to a different facility at any time du... = Yes

O1-24Bi Was there a specific reason for the individual's hospital transfer?

____
____
____
____
____

O1-24C Was the individual hospitalized in an Intensive Care or Critical Care Unit (ICU/CCU)?
- Yes
- No
- Unknown

O1-24D How long (days) was the individual's hospital stay?

____

O1-25 Was your family contacted by a health department following the individual's illness?

Yes

No

O1-26 Was an exposure questionnaire completed for the individual?

Yes

No

Display Question O1-26A:
If Was an exposure questionnaire completed for the individual? = Yes

O1-26A Who administered the questionnaire?

Self

Medical provider

Health department

Other (specify) ____

O1-27 Was there any other follow-up by a health department not previously discussed? If so, please describe.

____
____
____
____
____

O1-28 Which of the following conditions have developed following the individual's illness with HUS that were not previously occurring.

	Time since HUS				Was this condition formally diagnosed by a medical provider?			Are you currently taking medications for this condition?			Are you currently seeing a doctor for this condition?		
	6 months	1 year	5 year	10 year	Yes	No	Not Applicable	Yes	No	Not Applicable	Yes	No	Not Applicable
Intestinal problems (i.e., irritable bowel disease, inflammatory colitis, etc.)													
High blood pressure													
Heart disease													
Kidney failure (specify current % kidney function)													
Kidney transplant													
Other surgery (specify)													
Stroke													
Epilepsy / seizures													
Coma													
Bone disease or skeletal deformities													
Impaired growth													
Post-traumatic stress disorder													
Other (specify)													

SECTION "O" COULD BE REPEATED TO RECORD ILLNESS HISTORY FOR UP TO FIVE (5) OTHER INDIVIDUALS:

L3 I would like to:
- Complete illness history for another individual → REPEATS SECTION "O"
- Continue with the rest of the interview → CONTINUES WITH REST OF SURVEY

End of Block: History - Proxy

Start of Block: Quality of Life - interviewee

L8 The remainder of the interview will ask questions about your personal quality of life since the HUS event.

Q29 Have you experienced distressing memories about the HUS event?
- Yes
- No
- Unknown

Display Question Q29A:
If Have you experienced distressing memories about the HUS event? = Yes

Q29A Approximately how often do these memories bother you on a monthly basis?
- More than 6 times a month
- 4-6 times a month
- 2-3 times a month
- Less than 2 times a month
- Never

Q30 Have you experienced bad dreams or nightmares related to the HUS event?
- Yes
- No
- Unknown

Skip To: Q31 If Have you experienced bad dreams or nightmares related to the HUS event? != Yes

Q30A Do these dreams wake you up?
- Yes
- No
- Unknown

Q30B Are you able to return to sleep following these dreams?
- Yes
- No
- Unknown

Q31 Have you ever felt like you were experiencing the time immediately leading up to or following the HUS event again?

- Yes
- No
- Unknown

Display Question Q31A:
If Have you ever felt like you were experiencing the time immediately leading up to or following the... = Yes

Q31A Approximately how often do these flashbacks occur on a monthly basis?

- More than 6 times a month
- 4-6 times a month
- 2-3 times a month
- Less than 2 times a month
- Never

Q32 Have reminders of the HUS event caused you to become very EMOTIONALLY upset (i.e., fear, sadness, anger, guilt or shame, worry, etc.)

- Yes
- No
- Unknown

Q33 Have reminders of the HUS event caused you to become very PHYSICALLY upset (i.e., nausea, sweating, racing heart, shakiness, etc.)

- Yes
- No
- Unknown

Q34 Have you attempted to avoid thoughts or feelings related to the HUS event?

- Yes
- No
- Unknown

Q35 Have you attempted to make efforts to avoid activities, situations, or places that remind you of the HUS event or feel more dangerous since the HUS event?

- Yes
- No
- Unknown

Skip To: Q36 If Have you attempted to make efforts to avoid activities, situations, or places that remind you of... != Yes

Q35A What activities, situations, or places do you avoid?

____

Q35B What do you do to try and avoid these activities?

____

Q36 Is there any aspect of the time frame immediately leading up to the HUS event that you cannot remember (i.e., gap in memory)?

Yes
No
Unknown

Q37 Have you viewed yourself or the world in a more negative way since the HUS event?

Yes
No
Unknown

Display Question Q37A:
If Have you viewed yourself or the world in a more negative way since the HUS event? = Yes

Q37A What are some examples?

____
____
____
____
____

Q38 Have you blamed yourself for the HUS event and the ensuing outcomes?

Yes
No
Unknown

Display Question Q38A:
If Have you blamed yourself for the HUS event and the ensuing outcomes? = Yes

Q38A Approximately how often do you feel this way on a monthly basis?

More than 6 times a month
4-6 times a month
2-3 times a month
Less than 2 times a month
Never

Q39 Since the HUS event, have you lost interest in activities you used to participate in?

Yes
No
Unknown

Q40 Have you felt detached or cut off from others since the HUS event?
- Yes
- No
- Unknown

Display Question Q40A:
If Have you felt detached or cut off from others since the HUS event? = Yes

Q40A Approximately how many times do you feel this way on a monthly basis?
- More than 6 times a month
- 4-6 times a month
- 2-3 times a month
- Less than 2 times a month
- Never

Q41 Have you had difficulty experiencing positive feelings?
- Yes
- No
- Unknown

Display Question Q41A:
If Have you had difficulty experiencing positive feelings? = Yes

Q41A What are some examples?

____

Q42 Have you had difficulty concentrating?
- Yes
- No
- Unknown

Q43 Have you had difficulty falling or staying asleep?
- Yes
- No
- Unknown

Q44 How much have these difficulties been bothering you?
- A great deal
- A lot
- A moderate amount
- A little
- None at all

Q45 How much have these difficulties been interfering with your daily life?

- A great deal
- A lot
- A moderate amount
- A little
- None at all

Q46 Are there any other ways in which your experience with HUS has changed or impacted your daily life or lifestyle in any way?

____

Q47 Have you or any family members been formally diagnosed with post traumatic stress disorder (PTSD) or any other psychosocial disorders since the HUS event?

- Yes
- No
- Unknown

Display Question Q48:
If Have you or any family members been formally diagnosed with post traumatic stress disorder (PTSD)... = Yes

Q48 How many family members are experiencing these symptoms?

____

Q49 If you or anyone in your family would like additional information on health and well-being resources for managing these symptoms, you are welcome to contact the study coordinator who will work to put you in contact with the proper healthcare professionals.

End of Block: Quality of Life - interviewee
